# Data on a novel liver bioscaffold (rDLS) generated from regenerative liver with activated extracellular matrix for functional liver regeneration

**DOI:** 10.1016/j.dib.2018.07.021

**Published:** 2018-07-07

**Authors:** Wei Yang, Quanyu Chen, Lianhua Bai

**Affiliations:** a Hepatobiliary Institute, Southwest Hospital, Army Medical University, No. 30 GaotanYan, ShapingBa District, Chongqing 400038, China; b Key Laboratory of Freshwater Fish Reproduction and Development, Ministry of Education, Laboratory of Molecular Developmental Biology, School of Life Sciences, Southwest University, Beibei, Chongqing 400715, China

## Abstract

The data presented in this article are related to the original research article entitled “A novel bioscaffold with naturally-occurring extracellular matrix promotes hepatocyte survival and vessel patency in mouse models of heterologous transplantation” (Yang et al., in press) [1]. This article describes a decellularized liver scaffold (DLS) that derived from partial hepatectomy liver (rDLS) which supported primary hepatocyte survival and promoted blood patency, as compared with a conventional scaffold that generated from naïve liver (nDLS). Analysis by immunochemistry and scanning electron microscope (SEM) showed that the vessel network and extracellular matrix (ECM) components were similar to the nDLS. The rDLS could prevent blood clotting after transplanted it in vivo, identified by immunofluorescence staining for the integrin (αIIb, α4) expression and liver transplantation models (mice, pigs) a formed well-blood petency liver lobules. These data indicate that the novel scaffold (rDLS) with naturally-occurring “activated ECM” that may be useful for the implantation in vivo of a bioengineered organoid that is able to exert function long term without clotting in future clinic.

**Specifications Table**

**Table:** 

Subject area	Tissue engineering and regenerative medicine
More specific subject area	Biomaterials and regenerative medicine
Type of data	Supplementary figures
How data was acquired	Microscope, survey, scanning electron microscope (SEM), immunofluorescence staining, DNA extraction, surgical procedure
Data format	Raw, filtered, analyzed, etc
Experimental factors	Generation of rDLS by partial hepatectomy, identification of the rDLS Quality by SEM、ECM components immunochemistry (IHC), measurement for clotting by liver transplantation models (mouse, pigs)
Experimental features	Using software (SPSS 16.0) for data analysis
Data source location	Hepatobiliary Institute, Southwest Hospital, Army Medical University, Chongqing, China, 400038
Data accessibility	Data is within this article

**Value of the data**

• The data demonstrate for the first time that by using a novel approach to generate a decellularized liver scaffold (DLS) from regenerative liver (rDLS) which is different from the scaffold that generated from naïve livers (nDLS). The nDLS has been widely studies in the field.
• The data providing here for the first time support the rDLS with “active ECM environment” for preventing thrombosis in vivo, and the idea opens a new window for other researchers promising step toward to engineer functional organoids.

## 1 Data

**Fig. 1:**
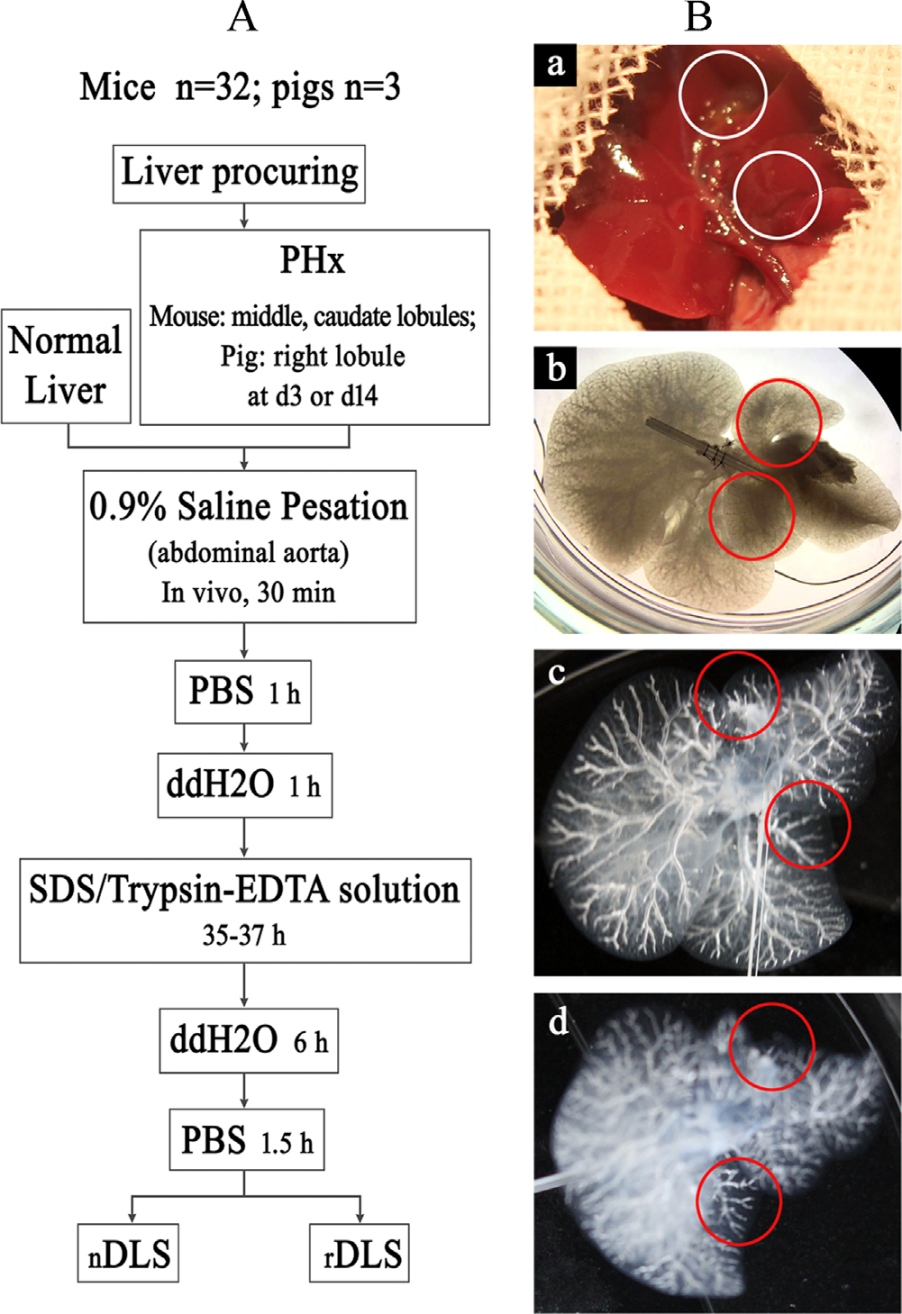
Generation of mouse natural decellularized liver scaffolds (DLS) from mouse partial hepatectomy tissue (rDLS) and normal liver (nDLS). (A) Description of the liver decellularization procedure. (Ba–d) Generation of the DLS was from normal liver (nDLS) and partial hepatectomy tissue (rDLS). Exposure of the liver for partial hepatectomy. Erythrocytes were removed from the liver via the abdominal aorta *in vivo* (a circles indicate the caudate and lobules). Both partial hepatectomy and normal liver tissue were removed from the body and perfused with SDS/Trypsin-EDTA solution. Perfusion was started through the portal vein. Representative photograph of the decellularized left lateral and median lobes of the mouse liver obtained after 35–37 h of perfusion, with the vascular tree visible from the nDLS (b; *n* = 30) and rDLS (c, d; circles indicate removed liver lobules; *n* = 32).

**Fig. 2:**
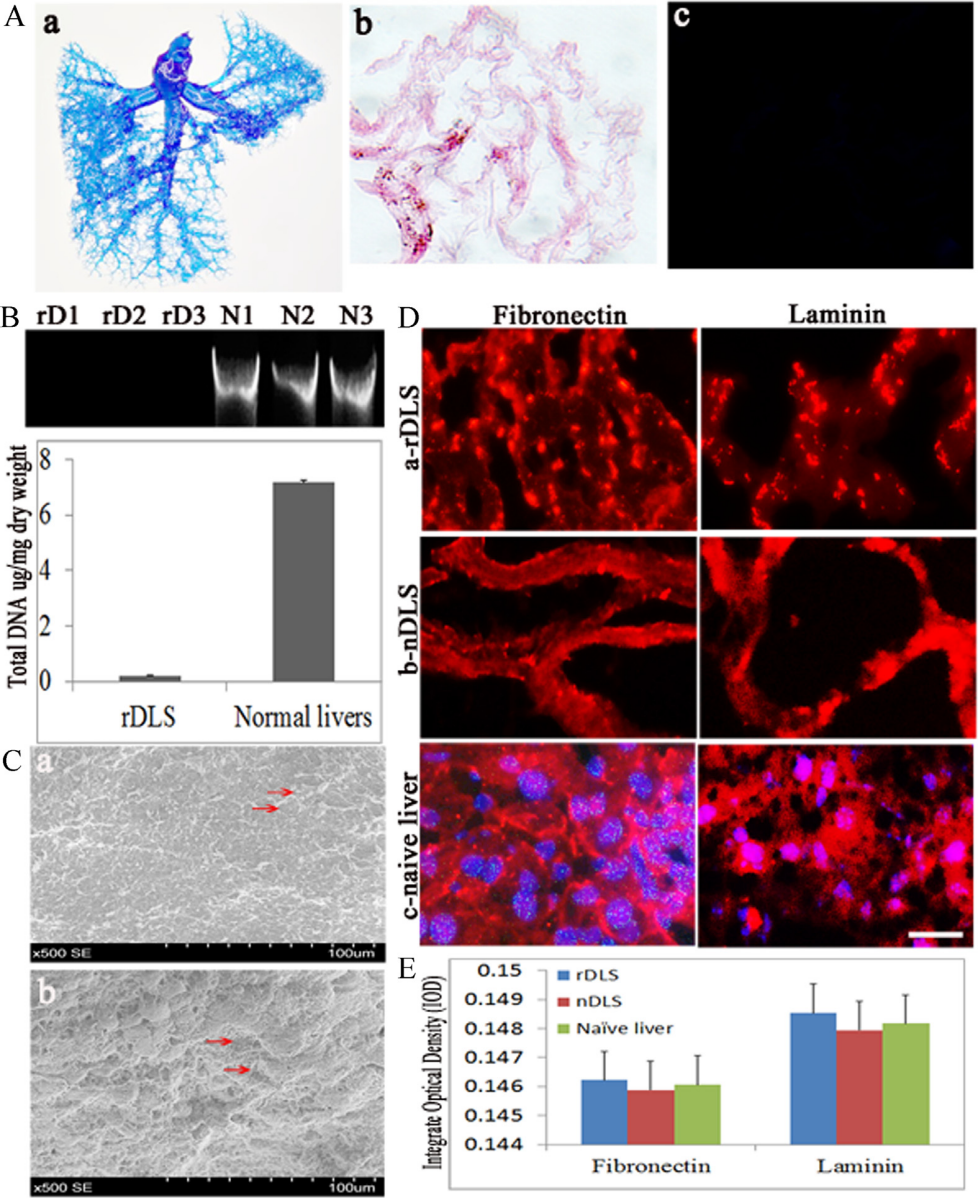
Characterization of the rDLS. A (a–c) The vascular tree of the rDLS after perfusion with Batson blue pigment dye (a) (*n* = 5), hematoxylin & eosin staining (b) (*n* = 10) and DAPI (4′,6-diamidino-2-phenylindole) staining (blue for nuclei) (c) (n = 10); the results indicated complete cell removal. Scale bar = 100 μm. B (a,b) DNA content of the rDLS (rD1–rD3) and nDLS (N1–N3) as determined with a PicoGreen Assay using a DNeasy Blood & Tissue Kit. Gels (a) and analysis (b) (rDLS: *n* = 11; naïve liver: *n* = 9). C (a,b) Scanning electron microscopy images of rDLS (*n* = 4) and naïve liver (*n* = 4) revealed a porous network structure in the scaffold that outlined the removed hepatocytes with preserved blood vessels (a, indicated by arrows) compared with the native liver that showed the presence of hepatocytes (b, indicated by arrows). D (a–c) The extracellular matrix (ECM) was evaluated by immunofluorescence. Fibronectin and laminin staining in rDLS (a), nDLS (b) and naïve liver (c). Red (Rhodamine) indicates specific staining for fibronectin and laminin. Blue: DAPI. Scale bar = 100 μm. (E) Quantification of fibronectin and laminin in the rDLS, nDLS and native liver. Data are shown as mean±standard deviation (SD) (*n* = 5 for each group). *P < 0.05 vs. nDLS.

**Fig. 3:**
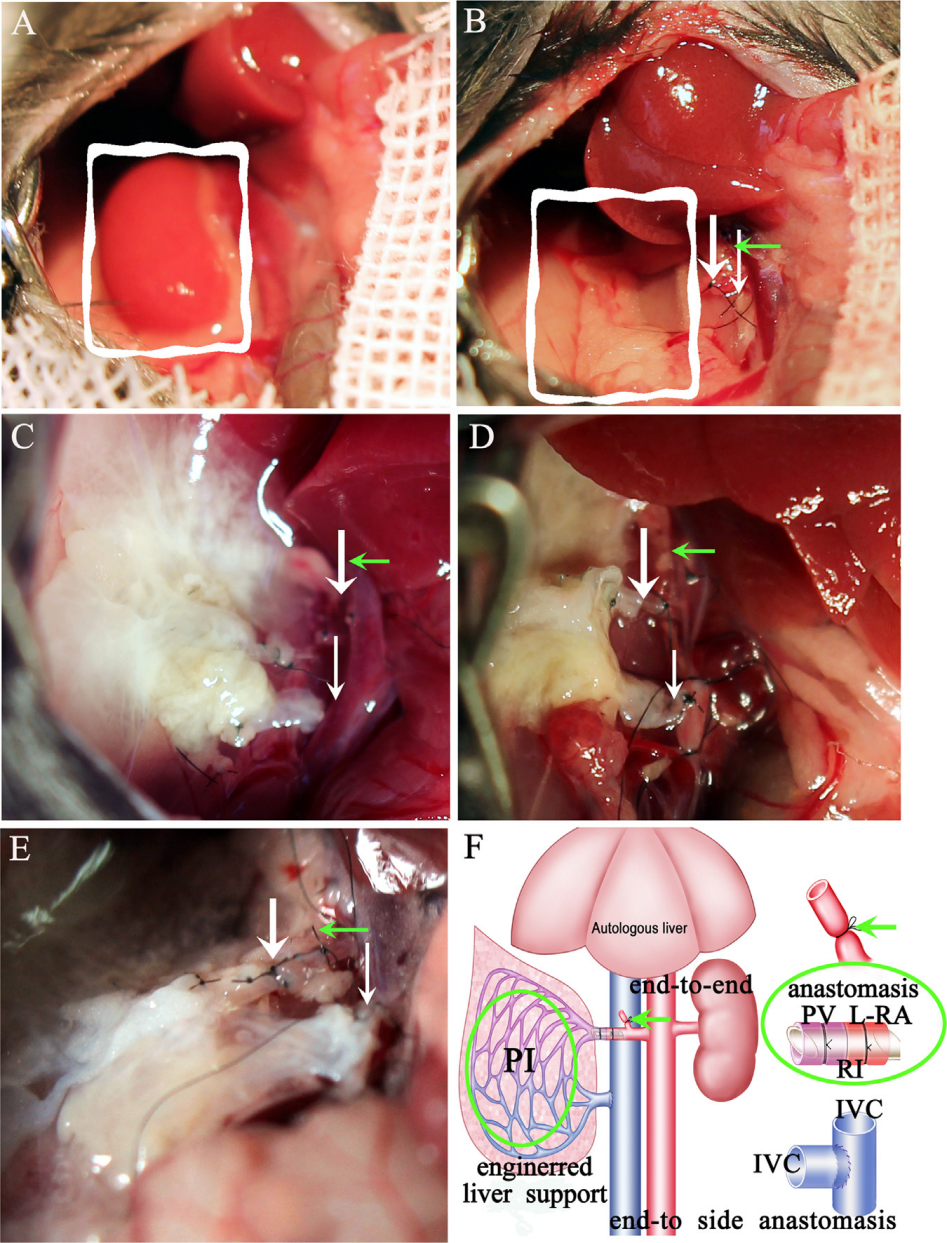
Portal-renal arterialized auxiliary heterotopic liver transplantation. (A) Exposure of the right-side kidney (the square indicates the kidney). (B) Nephrectomy of the right-side kidney (the square indicates the kidney removed). (C) The decellularized liver scaffold (DLS) was loaded with cells and positioned where the kidney had been removed. Bold arrow indicates the portal vein (PV), thin arrow indicates the inferior vena cava (IVC), green arrow indicates a right renal artery (Right-RA). The left-side renal artery (L-RA) was connected to the PV. Cross-clamping of the PV and IVC of the recellularized scaffold (D). The non-cell loaded DLS was connected to the recipient by the same procedure as the cell-loaded DLS, where the kidney had been removed (E). (F) Schematic of the operative procedure. The left green circle indicates the DLS. The right green circle indicates the end-end anastomosis between the PV (scaffold) and L-RA (recipient). The green arrows in the panels indicate the Right-RA. The right bottom schematic indicates the end-side anastomosis of the IVC (scaffold) and IVC (recipient).

**Fig. 4:**
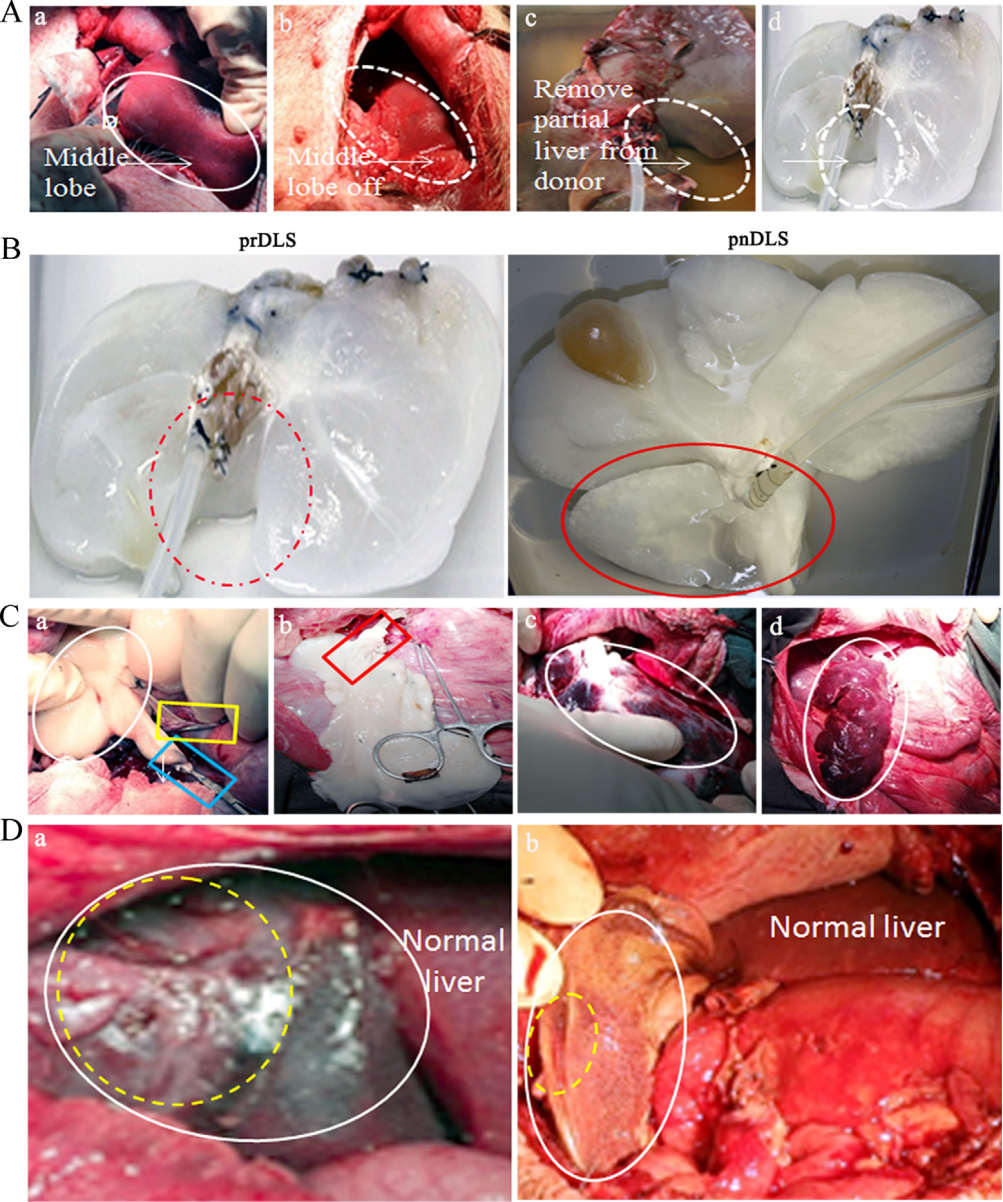
Detection of blood vessel patency in a decellularized liver bioscaffold generated from partial hepatectomy tissue from a large animal (pig) (prDLS) after auxiliary partial orthotopic liver transplantation (APOLT). (Aa–d) Procedure for prDLS generation (a); exposure and resection of liver middle lobule (b); middle lobule removal from the donor (c); and generation of an acellular scaffold by detergent perfusion for 24 h (d). (B) Representative photograph of the decellularization procedure from prDLS and normal porcine liver (pnDLS) after 24 h perfusion; the circles indicate middle lobule presence (b) and absence (a). (Ca–d) Procedure of APOLT for pDLS (a); donor pDLS-recipient connection via the portal vein-portal vein (indicated as a yellow square) and inferior vena cava-inferior vena cava (indicated as a blue square) via hepatic artery-hepatic artery connection (b, indicated as a red square); (c) blood reflow after the pDLS-recipient connection at 1 min (indicated as a circle) and 5 min (d, indicated as a circle). (D) Blood vessel patency in the prDLS (a) and pnDLS (b); white circles indicate the transplanted DLS; yellow circles indicate visible blood flow. (*n* = 3).

**Fig. 5:**
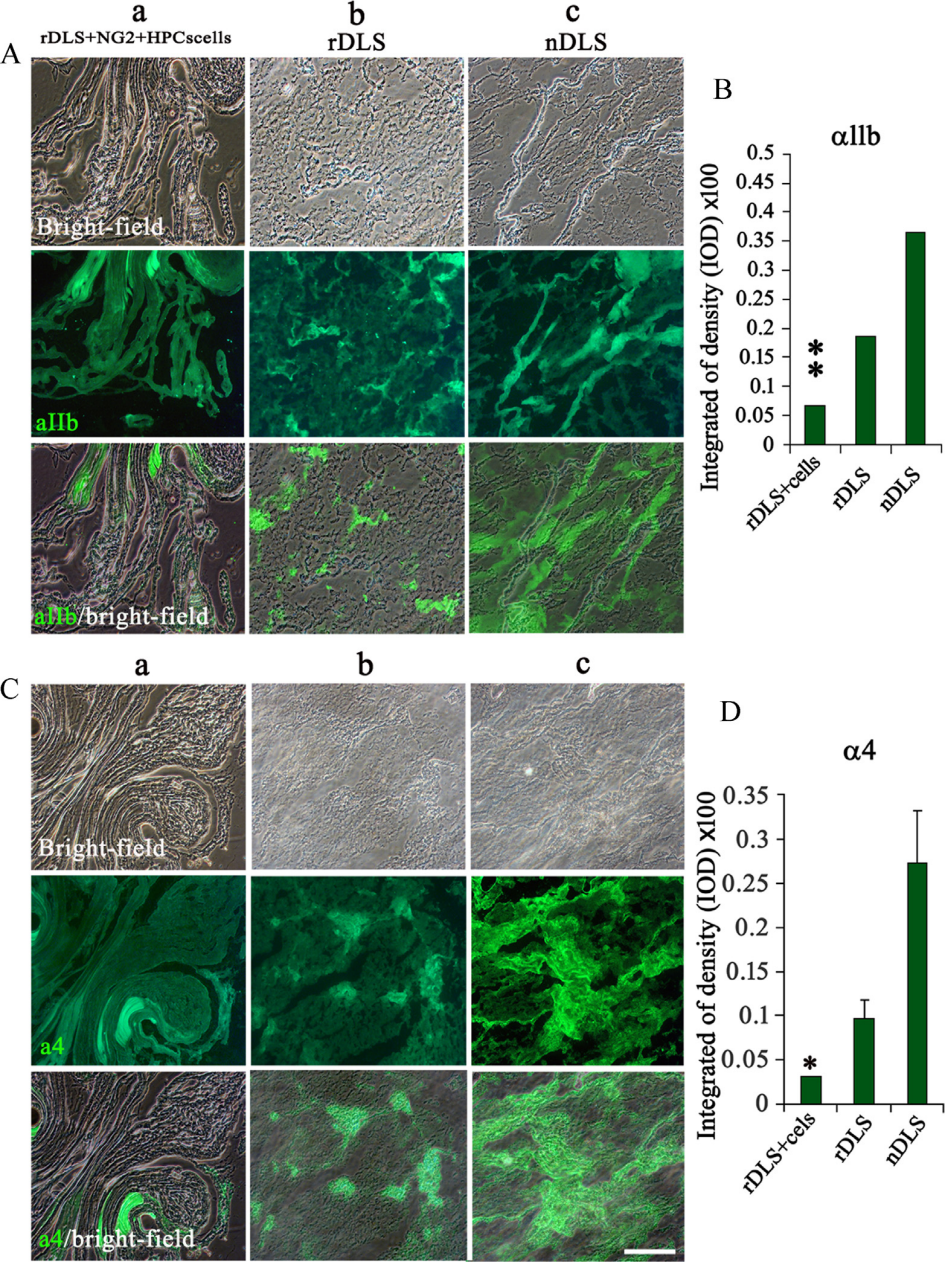
Detection of integrin expression 8 h after auxiliary heterotopic liver transplantation and reperfusion A (a–c) Expression of integrin αIIb (green) in rDLS (bright-field) loaded with NG2^+^HPCs (a), rDLS alone (b) and nDLS alone (c) and cultured for 7 days in endothelial cell conditioned medium (EC-CM). (B) Quantification of the integrin αIIb positive area (green) in the scaffolds from A (a–c). C (a–c) Expression of integrin α4 (green) in rDLS (bright-field) loaded with NG2^+^HPCs (a), rDLS alone (b) and nDLS alone (c) and cultured for 7 days with EC-CM. (D) Quantification of the integrin α4 positive area (green) in scaffolds from C (a–b). Scale bar = 100 μm. *n* = 3, *P < 0.05 and **P < 0.01 vs. nDLS.

**Fig. 6:**
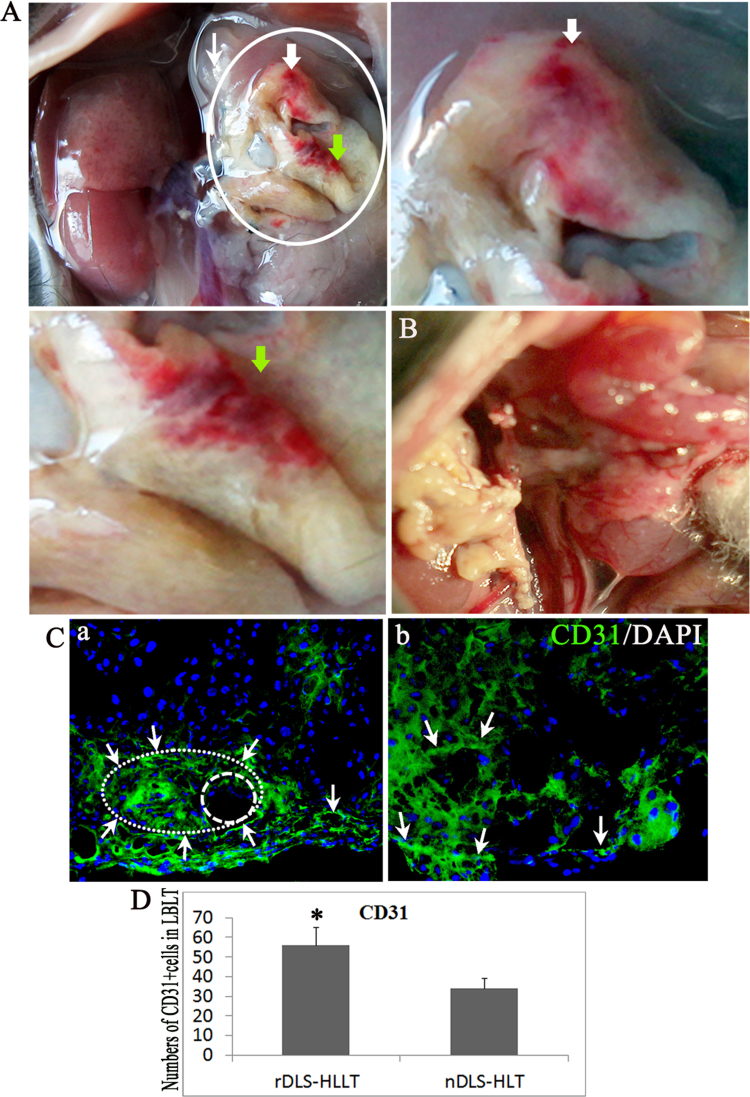
rDLS seeded with NG2^+^HPCs formed liver lobule-like tissue (LBLT) after auxiliary heterotopic liver transplantation. A (a–c) LBLT formed in rDLS loaded with NG2^+^HPCs for about 20–40 days (a, indicated as a circle), two lobes with better blood vessel patency (b, indicated by a white arrow; c indicated with a green arrow). (B) LBLT formed in nDLS loaded with NG2^+^HPCs for the same time with no visible blood flow (indicated as a circle) (*n* = 4). C (a,b) Well-aligned endothelial cells in rDLS-LBLT (a) compared to nDLS-LBLT (b), identified by immunofluorescence for CD31^+^ cells. (D) Quantification of CD31-positive cells (green) in rDLS-LBLT and nDLS-LBLT. Data are presented as mean ± SD from three independent experiments. Scale bar = 100 μm. *n* = 3, *P < 0.05 vs. nDLS-LBLT.

Naïve decellularized liver scaffold (nDLS)-based tissue engineering has been impaired by the lack of a suitable extracellular matrix (ECM) to provide “active micro-environmental” support [2]. Generation of the novel decellularized liver scaffold (rDLS) with “active micro-environmental” is shown in Fig. 1. We anesthetized mice with pentobarbital sodium salt and performed approximately 30–45% partial hepatectomy. The liver was perfused with detergent solution after three days [3] and the extracellular matrix was evaluated by SEM, DNA content, and immunofluorescence staining for ECM components (Fig. 2). The data indicate that rDLS has similar structure and ECM proteins compared to the nDLS. To address the anti-clotting effect in vivo, the rDLS was grafted into animals by portal-renal arterialized auxiliary heterotopic (mice) (Fig. 3) and inorthotropic (pig) (Fig. 4) liver transplantation. At this stage, the rDLS had lower expression of integrins (αIIb, α4) (Fig. 5) and higher blood patency than those of nDLS (Fig. 4). These are the first report on the anti-thrombosis of the rDLS. In addition, the rDLS/seed cell complex showed the better liver lobule formation after transplantation as compare to nDLS/seed cell complex in vivo (Fig. 6).

## 2 Experimental design, materials and methods

### 2.1 Generation of rDLS

The C57BL/6 mouse strain and pigs (China, Chongqing) was used in this study. A surgical method and perfusion procedure [4] was used for generation of rDLS. The Committee for the experiments involving animals care, handling and surgical procedures were performed in accordance with the guidelines of the Animal Care and Use Committee of the Third Military Medical University, Chongqing, China (Protocol no. SYXK-PLA-20120031).

### 2.2 DNA content measurement

DNA content was quantified as previously described [5]. In brief, the rDLS was digested with proteinase K (Invitrogen Inc., Carlsbad, CA, USA) at 37 °C and centrifuged at 2980g for 30 min. Supernatants were purified and centrifuged at 9000g for 30 min. Aqueous layers were removed and added to 3 M sodium acetate solution. Samples were treated with ethanol at − 20 °C for at least 8 h to precipitate the DNA quantified using the Picogreen DNA assay (Invitrogen Inc., Carlsbad, CA, USA), according to the manufacturer׳s instructions. DNA fragments were separated by electrophoresis on a 3% low melting point agarose gel with ethidium bromide at 60 V for 1 h and visualized under ultraviolet light (BioRad, Hercules, CA, USA).

### 2.3 Scanning electron microscopy

RDLS samples were assessed by scanning electron microscopy as previously described [6]. Briefly, rDLS samples were sectioned into small pieces (8 mm^3^), fixed in 4% glutaraldehyde stained with 1% osmium tetroxide (Electron Microscopy Sciences, Hatfield, PA, USA) for 1 h. Then, sections were washed in PBS and dehydrated using a graded series of alcohol. Critical drying was performed in absolute ethanol. Each sample was mounted on an aluminum stub and sputter-coated with a 7-nm layer of gold (Cressington 108 sputter coater, Cressington Scientific Instruments Ltd., Watford, UK) before examination using a JEM 6335F field emission scanning electron microscope (JEOL, Tokyo, Japan).

### 2.4 Immunofluorescence staining for platelet aggregation

The fixed DLS samples were embedded in flash-frozen in liquid nitrogen and sectioned to 5 μm. Sections were blocked with 10% fetal bovine serum for 30 min and primary antibodies (αIIb, α4, 1:100–500) were applied overnight at 4 °C. after secondary antibody, sections were mounted using fluorescence mounting medium (Dako, Glostrup, Denmark). Fluorescence signals were observed under a fluorescence microscope (SMZ25/SMZ18; Nikon, Tokyo, Japan). Analysis was performed using Image J software (National Institutes of Health, Bethesda, MI, USA).

### 2.5 Surgical mouse and pig model for thrombosis measurement

Mice were anesthetized and opened along the midline to expose the right kidney. After clamping the right branches of the renal artery and vein, a nephrectomy was performed. Once the right kidney was removed, the DLS was placed into the nephrectomy site. The portal vein and inferior vena cava of the host were introduced into the DLS graft׳s portal vein, and another of the same size was introduced and secured in the inferior vena cave. An end-to-end anastomosis was made between the cuff of the recipient׳s left renal artery branch and the scaffold׳s portal vein. The connection of the recipient׳s inferior vena cava to that of the scaffold was performed using end-to-side anastomosis and Any blood lost during surgery was replaced with lactated Ringer׳s solution.

## References

[1] Yang W., Chen Q., Xia R. (2018). A novel bioscaffold with naturally-occurring extracellular matrix promotes hepatocyte survival and vessel patency in mouse models of heterologous transplantation. Biomaterial.

[2] Perniconi B., Costa A., Aulino P. (2011). The pro-myogenic environment provided by whole organ scale acellular scaffolds from skeletal muscle. Biomaterial.

[3] Kono H., Fujii H., Suzuki-Inoue K. (2017). The platelet-activating receptor C-type lectin receptor-2 plays an essential role in liver regeneration after partial hepatectomy in mice. J. Thromb. Haemost. J. Thromb. Haemost..

[4] Zhang H., Zhang Y., Ma F. (2015). Orthotopic transplantation of decellularized liver scaffold in mice. Int. J Clin. Exp. Med..

[5] Gilbert T.W., Freund J.M., Badylak S.F. (2009). Quantification of DNA in biologic scaffold materials. J. Surg. Res..

[6] Soto-Gutierrez A., Zhang L., Medberry C. (2011). A whole-organ regenerative medicine approach for liver replacement. Tissue Eng. Part C Methods.

